# Polysaccharide-Derived Drug Delivery Systems: Therapeutic Potential in Rheumatoid Arthritis—A Review

**DOI:** 10.3390/ph19101545

**Published:** 2026-09-29

**Authors:** Xiaotong Yue, Aishuo Yu, Jinjin Chen, Feilong Zhao, Liyang Zhang, Abudureheman Bahatibieke, Yudong Zheng, Wei He, Jin Xu

**Affiliations:** 1 School of Materials Science and Engineering, University of Science and Technology Beijing, Beijing 100083, China; 15092648529@163.com (X.Y.); m17866920727@163.com (A.Y.); m202510282@xs.ustb.edu.cn (J.C.); zhaoofeilong@163.com (F.Z.); zly-0504@163.com (L.Z.); b20200193@ustb.edu.cn (A.B.); zhengyudong@master.ustb.edu.cn (Y.Z.); 2 Department of Basic Medicine, Kangda College of Nanjing Medical University, Lianyungang 222000, China

**Keywords:** polysaccharide derivatives, rheumatoid arthritis, drug delivery, stimuli-responsiveness

## Abstract

Rheumatoid arthritis (RA) is a systemic autoimmune disease characterized by chronic synovial inflammation, synovial hyperplasia, and progressive joint destruction and remains a major clinical challenge. Polysaccharide-derived delivery platforms have attracted widespread attention for RA precision therapy owing to their natural origin, excellent biocompatibility, and tailorable functionality; however, existing reviews generally focus on single material or carrier types and provide limited cross-comparison of carrier architectures and translational considerations. This review systematically evaluates recent advances in polysaccharide derivative design strategies and delivery system innovations for RA management. We first elaborate on how chemical modifications confer multi-responsive properties (pH-, reactive oxygen species [ROS]-, and enzyme-triggered release) and how diversified morphologies (hydrogels, microspheres, microneedles, vesicles) enable spatiotemporally controlled drug delivery in inflamed joints. Critical analysis highlights several translational barriers, including limited in vivo retention, insufficient pharmacokinetic and biodistribution data, and challenges associated with scale-up manufacturing and reproducibility. Looking forward, future development should integrate biomimetic design, rational multi-component optimization, and advanced manufacturing technologies to improve the stability, controllability, and translational feasibility of polysaccharide-based RA delivery systems. This review provides an integrated, translation-focused perspective that bridges material engineering and clinical pathological demands, guiding the development of next-generation polysaccharide-based RA therapies.

## 1. Introduction

Rheumatoid arthritis (RA) is a systemic autoimmune disease characterized by persistent synovial inflammation, joint swelling, and progressive cartilage and bone damage [1]. Current treatment follows a treat-to-target strategy and includes conventional synthetic disease-modifying antirheumatic drugs (csDMARDs), biological DMARDs (bDMARDs), and targeted synthetic DMARDs (tsDMARDs). Methotrexate (MTX) remains a cornerstone of initial treatment, while tumor necrosis factor (TNF) inhibitors, interleukin-6 (IL-6) pathway inhibitors, and B-cell-targeted therapies such as rituximab provide additional options for patients with inadequate response to conventional treatment. Janus kinase (JAK) inhibitors have further expanded the therapeutic landscape. Nonsteroidal anti-inflammatory drugs (NSAIDs) and glucocorticoids remain useful for symptomatic or short-term inflammatory control but are not substitutes for disease-modifying therapy [2,3,4]. Despite these advances, incomplete treatment response, adverse effects, and the need for long-term administration remain important challenges, supporting continued investigation of delivery strategies that may improve local drug exposure while limiting systemic burden.

In recent years, localized drug delivery systems (DDS) based on polysaccharide derivatives have emerged as a research focus in RA treatment, owing to their favorable biocompatibility, biodegradability, and microenvironment-responsive properties. Natural polysaccharides—such as chitosan (CS), hyaluronic acid (HA), and dextran—are obtained from diverse sources (including animals, plants, and microorganisms) [5]. Their derivatives can be chemically modified (e.g., via hydrophobic modification or conjugation with targeting ligands) to endow the carriers with smart responsive capabilities (e.g., pH-, ROS-, or enzyme-triggered release), thereby enabling precise spatiotemporal regulation of drug distribution within inflamed joints [6]. Moreover, the variety of carrier forms (e.g., hydrogels, microspheres, microneedles) further enhances adaptability to different therapeutic scenarios: injectable hydrogels facilitate sustained release, transdermal microneedles allow non-invasive administration, and nanoparticles are suitable for targeted gene therapy delivery [7,8,9,10].

Despite growing research interest in this field, existing review articles present notable gaps that motivate the present work. First, most reviews are confined to narrow scopes: Kumar et al. focused exclusively on natural polysaccharide nanoparticles for RA [11], while Walvekar et al. limited their discussion to HA-based carriers for arthritis management [12]. These works cannot provide a cross-categorical comparative perspective across different carrier architectures. Second, prior reviews predominantly adopt a material-centric approach, describing carrier types in isolation without aligning them to the dynamic pathological stages of RA. This disconnects material design from clinical therapeutic demands, hindering translational interpretation. Third, existing reviews largely catalog published studies descriptively, with limited critical analysis of methodological trade-offs, translational bottlenecks, and comparative performance across systems. To address these gaps, this review adopts a comparative and translation-oriented framework to systematically evaluate hydrogels, nanoparticles, microneedles, and composite delivery systems. Rather than assigning specific carrier types to predefined disease stages, we critically assess their therapeutic applicability, technical merits, limitations, and translational challenges according to drug properties, administration routes, release characteristics, and pathological context. Rather than simply enumerating published works, we critically assess their therapeutic applicability, technical merits, and limitations across acute and chronic RA management. We argue that the future of polysaccharide therapies lies not in universal carriers but in rational design strategies that match drug release kinetics, stimulus responsiveness, and administration routes to the dynamic characteristics of the disease. By integrating recent advances in polysaccharide engineering and multi-stimulus responsive systems, this paper aims to provide a more integrated, critical, and forward-looking perspective—bridging material design with pathological and clinical demands to guide the development of next-generation therapeutic solutions.

## 2. Literature Search and Selection Strategy

A structured literature search was conducted to identify studies relevant to polysaccharide-based and polysaccharide-derived drug delivery systems for rheumatoid arthritis (RA). PubMed, Web of Science Core Collection, Scopus, and ScienceDirect were searched using combinations of terms related to RA, polysaccharides, and drug delivery. The main search terms included “rheumatoid arthritis,” “inflammatory arthritis,” “polysaccharide,” “polysaccharide derivative,” “hyaluronic acid,” “chitosan,” “chondroitin sulfate,” “dextran,” “alginate,” “nanoparticle,” “hydrogel,” “microsphere,” “microneedle,” “vesicle,” “drug delivery,” “targeted delivery,” and “stimuli-responsive delivery.” The search covered publications from 2010 to 2026, with emphasis on recent studies.

Studies were included when they investigated polysaccharide-based, polysaccharide-derived, or polysaccharide-containing delivery systems and provided relevant information on carrier design, drug delivery, therapeutic mechanisms, or biological evaluation in RA, inflammatory arthritis, or related inflammatory models. Studies conducted in osteoarthritis (OA) were retained only when they provided transferable information on carrier design or delivery mechanisms, and such evidence is explicitly identified as OA evidence in the revised manuscript. Studies were excluded when they were unrelated to drug delivery, lacked a meaningful polysaccharide-related function, were duplicate publications, or were conference abstracts, editorials, and other non-primary research articles.

Because the purpose of this review is critical comparison rather than quantitative meta-analysis, the selected studies were analyzed according to carrier architecture, polysaccharide function, therapeutic cargo, administration route, disease model, targeting mechanism, release characteristics, therapeutic outcome, safety evaluation, and translational relevance.

## 3. Pathogenesis and Therapeutic Needs of Ra

RA represents a systemic autoimmune disorder distinguished by symmetrical polyarticular chronic synovitis, sustained by pathogenic autoantibodies including anti-citrullinated protein antibodies (ACPA) and rheumatoid factor (RF) [13,14]. These immunologic drivers facilitate progressive articular destruction and systemic inflammatory manifestations through multifaceted mechanisms.

**Figure 1:**
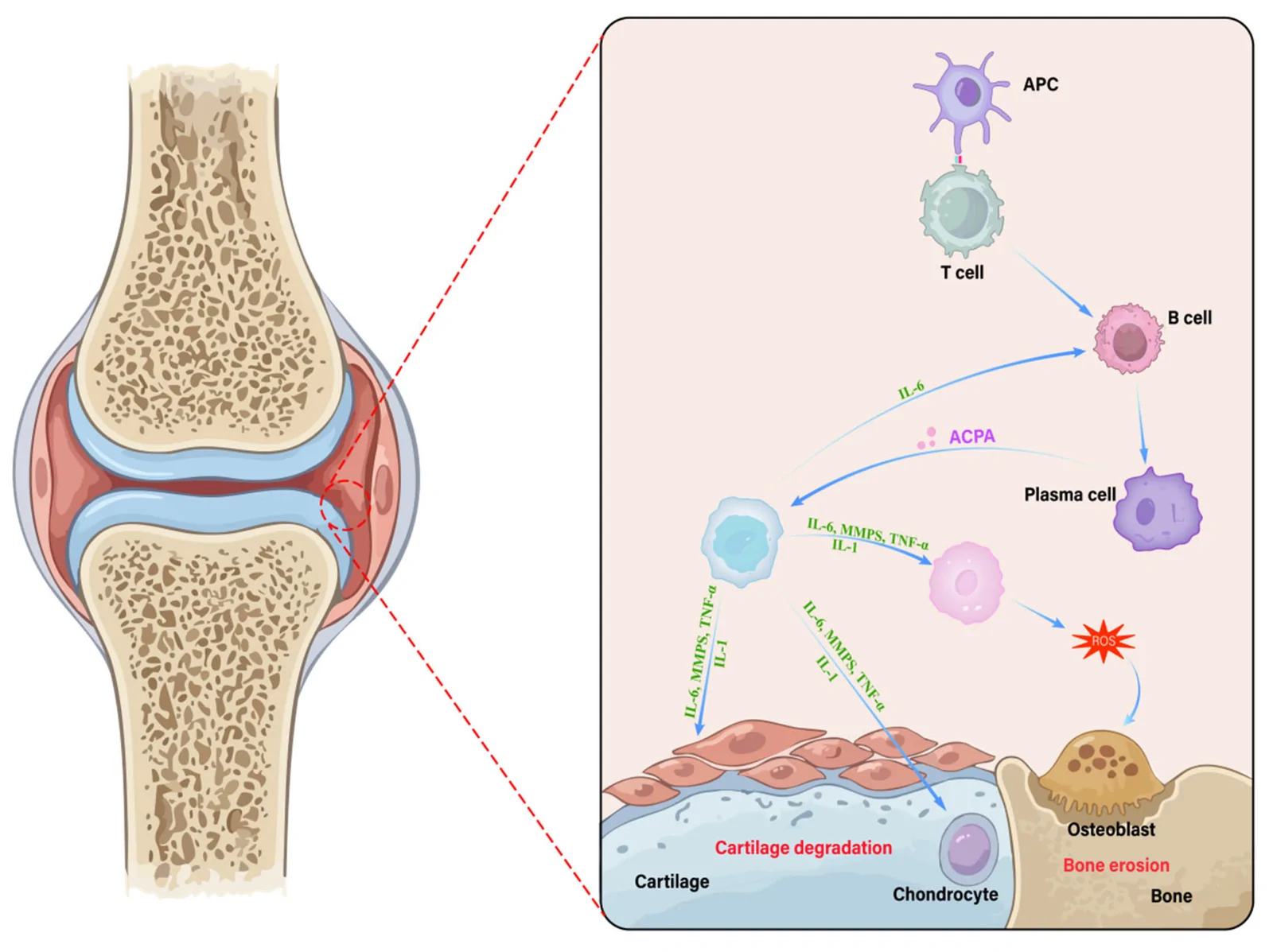
Pathogenesis of RA. Different colors and symbols represent the indicated immune cells, joint-resident cells, and inflammatory mediators; arrows indicate the direction of immune and inflammatory interactions. APC, antigen-presenting cell.

The disease pathogenesis involves a tripartite interaction of genetic predisposition (notably HLA-DRB1 alleles containing shared epitopes), environmental exposures (including tobacco use and microbial triggers such as Porphyromonas gingivalis), and immune homeostasis disruption [15]. The emergence of ACPA and anti-modified protein antibodies (e.g., anti-carbamylated proteins) signifies critical breakdown of self-tolerance, initiating autoreactive B-cell differentiation and subsequent T-cell activation. Th17 lymphocytes coordinate synovial fibroblast (FLS) activation through IL-17 secretion, synergizing with macrophage-derived cytokines (TNF-α, IL-6) to establish a self-perpetuating inflammatory milieu [16]. These interactions collectively amplify the inflammatory cascade through positive feedback mechanisms. The abnormal proliferation and metabolic reprogramming of FLS—characterized by enhanced glycolysis—further perpetuate inflammation and ultimately contribute to cartilage destruction and bone erosion via the secretion of matrix metalloproteinases (MMPs) [17]. Figure 1 shows the pathogenesis of RA. Meanwhile, oxidative stress, ferroptosis, and H-type angiogenesis promote sustained inflammatory responses and disrupt bone metabolic balance within the joint microenvironment [18,19].

From a drug delivery design perspective, these pathological features directly define three core requirements for carrier development: first, the multi-cellular inflammatory cascade necessitates carriers capable of co-delivering agents targeting multiple pathological nodes; second, the synovial barrier and rapid lymphatic clearance require carriers with enhanced tissue retention and deep penetration capacity; third, the fluctuating disease course between acute flares and chronic remission calls for tunable release profiles rather than a uniform delivery pattern [20].

Currently, no cure exists for RA, and the core objective of treatment remains interrupting the vicious cycle of “inflammation–destruction.” NSAIDs alleviate inflammation by inhibiting the cyclooxygenase (COX) pathway, yet long-term use often leads to gastrointestinal mucosal injury and cardiovascular complications [21]. Glucocorticoids rapidly suppress synovial inflammation, but repeated administration may result in chondrocyte apoptosis and disrupted bone metabolism. Conventional disease-modifying antirheumatic drugs (DMARDs) modulate immune responses; however, they pose risks of hepatotoxicity, nephrotoxicity, and bone marrow suppression. Regarding administration routes, oral and intravenous systemic delivery represent conventional approaches for RA management. Nevertheless, drugs exhibit poor joint accumulation, are rapidly cleared, and often require frequent dosing due to subtherapeutic local concentrations. Furthermore, adverse effects in non-target organs remain an inherent drawback of systemic administration [22]. Intra-articular administration can increase drug exposure within a selected joint and is therefore attractive for localized therapy. However, the intra-articular fate of therapeutic agents depends on multiple factors, including molecular size and charge, formulation properties, synovial permeability, cartilage structure, joint inflammation, lymphatic drainage, and the physicochemical properties of the formulation [20,23]. These factors influence synovial retention, tissue penetration, cellular uptake, and clearance, and therefore intra-articular residence time varies substantially among different therapeutic agents and experimental conditions. Consequently, fixed values for synovial pore size or intra-articular half-life should not be considered universal design thresholds. Instead, delivery systems should be designed according to the properties of the therapeutic cargo, the target tissue, and the desired duration of local exposure.

Over the long term, these conventional treatments often fail to yield satisfactory outcomes, which may be attributable to pronounced systemic toxicity or limited therapeutic duration. Hence, overcoming the drawbacks of traditional carriers by developing long-acting and biocompatible drug delivery systems that target the joint microenvironment and enable precise, controlled release has become a pivotal research direction in RA therapy.

## 4. Derivatives of Polysaccharides

**Figure 2:**
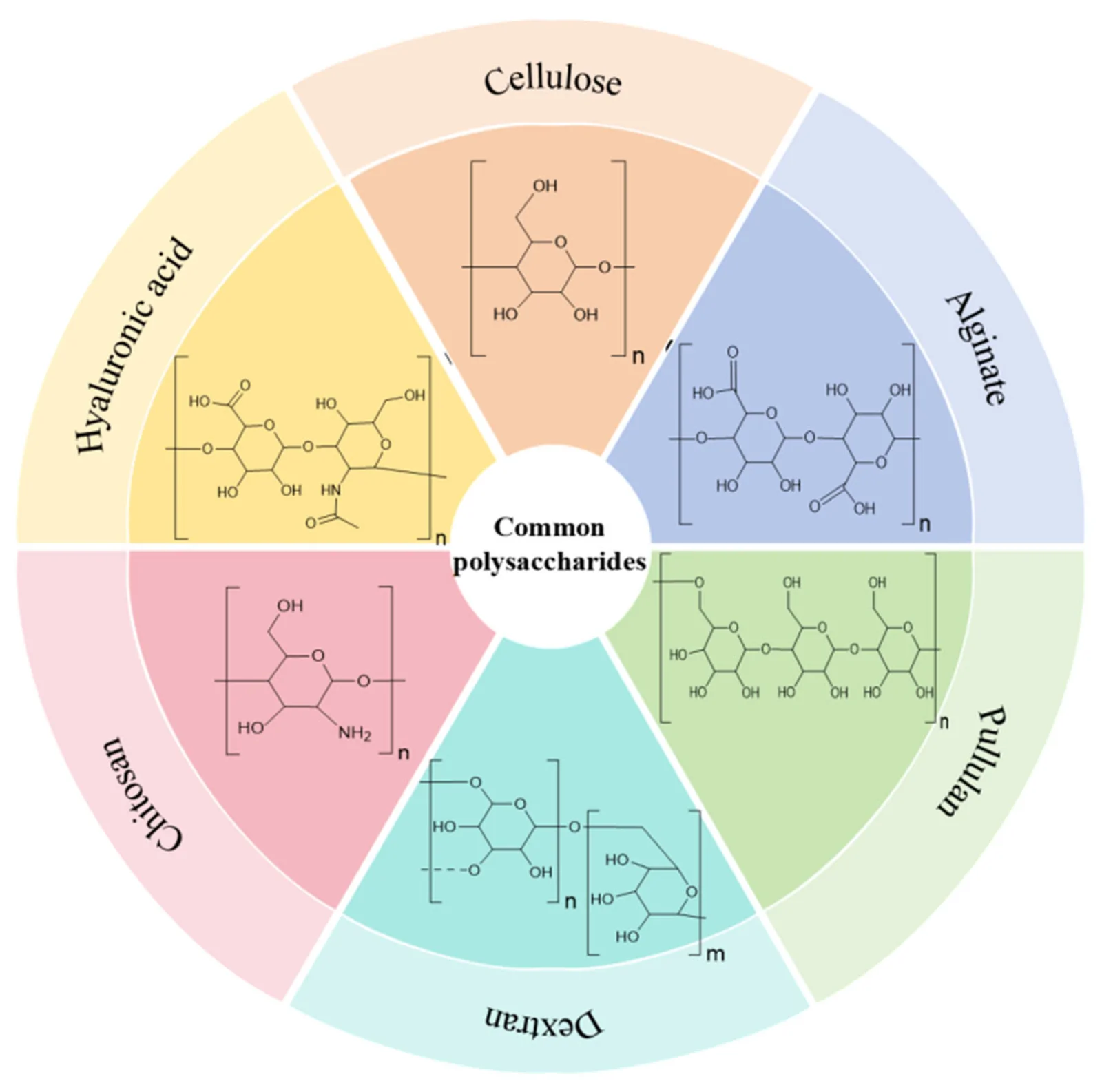
Common polysaccharides used for fabrication of RA drug delivery systems.

Polysaccharides are high-molecular-weight polymers formed through the polymerization of monosaccharides via glycosidic bonds. They display diverse architectures, including linear or branched chain conformations, and can give rise to distinct polysaccharide structures through various glycosidic linkages, such as α-1,4, β-1,4, and α-1,6 [23]. These polysaccharides are found in a wide range of organisms, including plants, animals, and microorganisms. Examples include cellulose from plants, hyaluronic acid (HA) as a glycosaminoglycan found in vertebrate tissues, chondroitin sulfate (CS) from animal tissues and other biological sources, and dextran produced primarily by microorganisms. Chitosan is obtained by deacetylation of chitin, which is mainly sourced from crustacean shells but can also be obtained from fungi and insects [24]. The abundance of reactive groups, such as hydroxyl groups, amino groups, and others, present in the molecular chains of these polysaccharides provides a structural basis for chemical modification [25]. Through the implementation of chemical modification strategies, such as sulfation, carboxymethylation, or graft copolymerization, natural polysaccharides can be transformed into derivatives with targeted delivery and microenvironmental response properties while retaining their inherent biocompatibility and degradability. This renders them ideal candidates for use in RA drug delivery systems [26]. Polysaccharides are found in abundance in the biological world. They are found in a variety of sources, including plants (e.g., starch, pectin), animals (e.g., chitin, HA, and heparin), microorganisms (e.g., dextran), and algae (e.g., alginate) [27]. Figure 2 shows common polysaccharides used for the fabrication of RA drug delivery systems: (a) HA; (b) cellulose; (c) alginate; (d) CS; (e) dextran; (f) pullulan. Polysaccharide derivatives exhibit a distinct advantage in terms of functional programmability, which refers to the ability to selectively modify material properties through precise chemical modification and structural design.

The distinct structural features of polysaccharides dictate their diverse physicochemical properties, laying a foundation for subsequent polysaccharide design. Certain polysaccharides inherently possess exceptional characteristics: HA, for instance, is a natural extracellular matrix component that exhibits intrinsic lubricating and anti-inflammatory functions while targeting CD44 receptors [28]. CS is widely utilized owing to its biodegradability and antimicrobial activity [29]. These inherent advantages position polysaccharides and their derivatives as highly promising materials for biomedical applications, particularly in chronic conditions such as RA that demand long-term, localized therapeutic strategies. For clarity, this review distinguishes polysaccharide-derived systems from broader polysaccharide-containing systems according to the functional contribution of the polysaccharide component. Polysaccharide-derived systems primarily refer to carriers in which a polysaccharide or chemically modified polysaccharide forms the main carrier framework. Polysaccharide-based systems are used more broadly for delivery platforms in which polysaccharides provide an essential structural or functional component. By contrast, polysaccharide-containing composite systems include substantial non-polysaccharide components, such as PLGA, graphene oxide, carbon dots, or metallic nanomaterials, while the polysaccharide may serve as a carrier matrix, surface coating, targeting ligand, or responsive component. This distinction is used throughout the revised manuscript to avoid attributing the performance of a hybrid system solely to its polysaccharide component.

## 5. Polysaccharide-Based Drug Delivery Systems

Polysaccharide-based drug delivery platforms have emerged as multifaceted therapeutic candidates for RA intervention, with their therapeutic superiority stemming from intrinsic bio-origin, demonstrated hemocompatibility, and tunable chemical functionality. Through rational structural engineering guided by carrier morphology optimization and pathophysiological requirements, polysaccharide derivatives can be precisely tailored into diverse therapeutic architectures, including, but not limited to, stimuli-responsive hydrogels, transdermal microneedle arrays, and nanoscale particulate systems, each offering distinct spatiotemporal control over drug bioavailability [30,31,32]. The drug delivery process focuses on five performance aspects: circulation, accumulation, permeation, internalization, and release [33]. Each aspect critically influences delivery efficacy. Consequently, individual carriers offer distinct advantages for RA treatment.

### 5.1. Polysaccharide-Based Hydrogel

Hydrogels represent a class of highly hydrophilic three-dimensional network polymers characterized by rapid water absorption and swelling capacity, exhibiting physicochemical properties closely resembling those of natural soft tissues. Hydrogels are highly hydrophilic three-dimensional networks that can provide a hydrated environment and tunable mechanical and transport properties. In RA, persistent synovial inflammation and altered extracellular matrix remodeling create challenges for maintaining therapeutic agents within the joint. Injectable hydrogels have therefore been investigated as local depots that can prolong drug retention and regulate release through control of network density, cross-linking chemistry, degradation, and matrix–drug interactions [8,20,34]. Evidence from OA models is discussed separately when it provides transferable information on carrier design or intra-articular delivery, but such findings are not considered direct evidence of therapeutic efficacy in RA.

**Figure 3:**
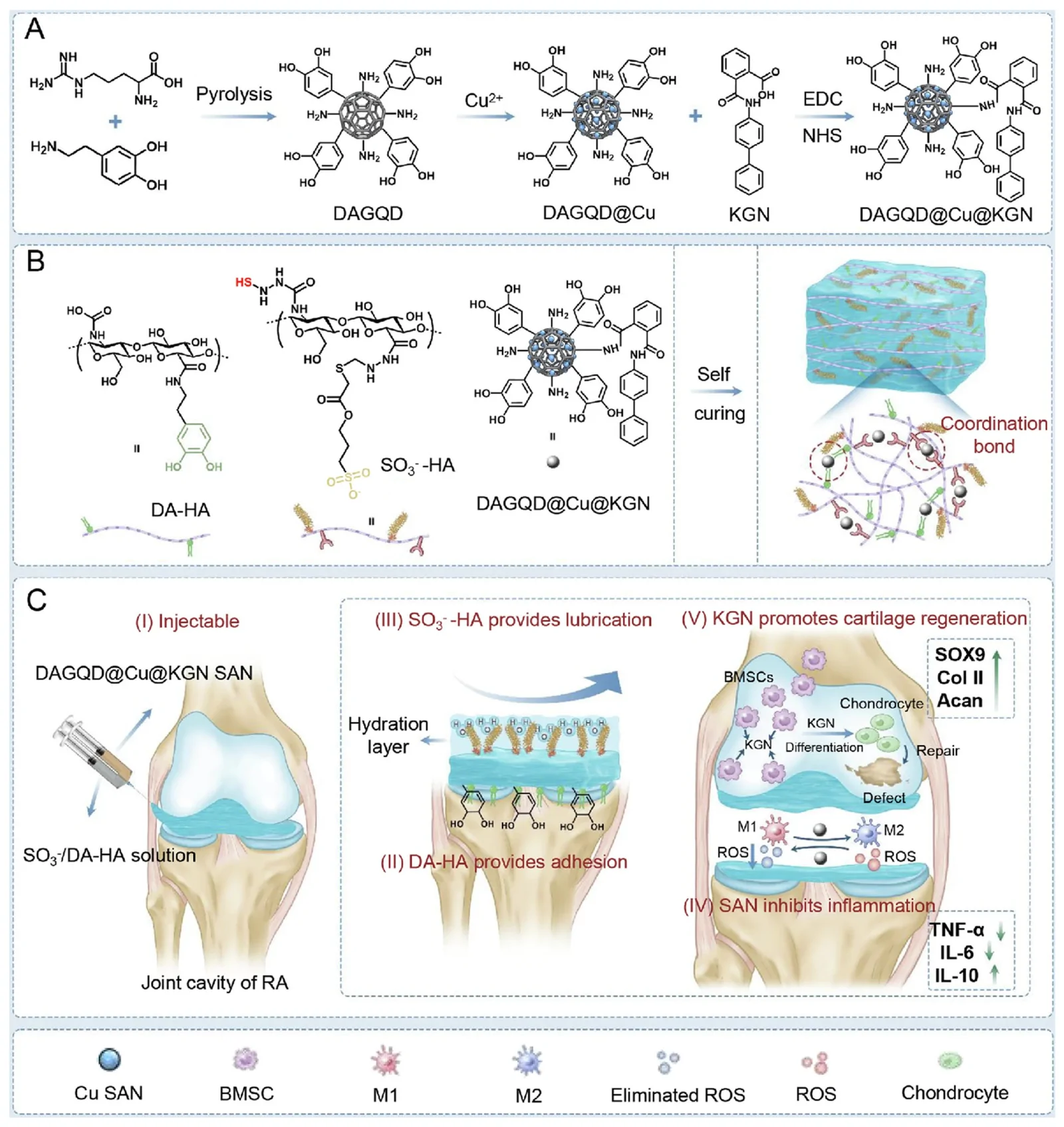
Synthesis of DAGQD@Cu SAN, DAGQD@Cu@KGN SAN and DAGQD@Cu@KGN-SO3−/DA-HA hydrogel. (**A**) Synthesis of DAGQD@Cu SAN and DAGQD@Cu@KGN SAN. (**B**) Synthesis of DAGQD@Cu@KGN-SO_3_^−^/DA-HA hydrogel. (**C**) DAGQD@Cu@KGN-SO_3_^−^/DA-HA hydrogel for RA therapy. (**I**) Hydrogel self-cures in situ within the joint cavity; (**II**,**III**) DAGQD@Cu@KGN-SO3−/DA-HA hydrogel displays lubrication and adhesion properties; (**IV**) regulation of inflammatory microenvironment facilitated by SAN with remarkable ROS-scavenging capabilities; (**V**) KGN released from the hydrogel promotes the recruitment of BMSCs and induces their differentiation into chondrocytes, facilitating the repair of cartilage damaged by RA. Arrows indicate the synthesis, self-curing, and biological processes, while different colors and symbols represent Cu SAN, BMSCs, M1/M2 macrophages, ROS, eliminated ROS, and chondrocytes, as indicated in the figure legend. Reproduced from Ref. [37] under the Creative Commons Attribution-NonCommercial-NoDerivatives 4.0 International License.

Although polysaccharides offer favorable biocompatibility, natural origin, and abundant sourcing, they still exhibit limitations in certain complex applications—such as insufficient mechanical strength and lack of inherent responsiveness. Consequently, research efforts increasingly concentrate on polysaccharide-derived hydrogels. By leveraging the structural advantages of polysaccharides, researchers employ chemical modifications and controlled cross-linking to precisely regulate charge density, hydrophilicity/hydrophobicity, molecular conformation, and interaction sites. This strategy aims to overcome the inherent drawbacks of natural polysaccharides and enable tailored design of material properties [35]. A representative study by Xu et al. demonstrated this paradigm through fabricating a temperature-responsive self-healing hydrogel (OCF/siHMGB1) via imine bond-mediated crosslinking between CS and oxidized chondroitin sulfate (OCS), supplemented with β-glycerophosphate sodium. This innovative system achieved 2.3-fold greater mechanical resilience compared to conventional CS/β-GP formulations while concurrently addressing two critical challenges: siRNA molecular instability and inadequate intra-articular retention duration. Notably, the hydrogel effectively induced macrophage phenotype transition from pro-inflammatory M1 to regenerative M2 polarization [36]. Recent advances have further incorporated nanotechnology to enhance therapeutic efficacy. He and colleagues developed a copper single-atom nanoenzyme hydrogel (DAGQD@Cu@KGN) to improve drug delivery efficiency [37] (Figure 3). This system mimics synovial fluid functionality using sulfated hyaluronic acid (SO_3_^−^-HA) and achieves rapid in situ gelation via Cu2+ coordination cross-linking with dopamine-modified hyaluronic acid (DA-HA). By integrating targeted receptors with nanozyme activity, the hydrogel synergistically enhances ROS scavenging and supports cartilage regeneration [38]. Further advancing the field, Liu and colleagues developed a ROS/pH dual-responsive hydrogel system (HC@PTM) incorporating thioredoxin-mediated micellar drug reservoirs. This design achieved spatiotemporally controlled drug release coupled with dynamic free radical elimination, exemplifying the convergence of precision delivery and pathological microenvironment remodeling strategies.

With the progression of fundamental research and clinical requirements, multifunctional integrated design has become a pivotal strategy for innovating beyond traditional carrier frameworks. The thermosensitive Epimedium polysaccharide hydrogel (OFP-CS-F407-MTX) developed by Zhang’s team used Schiff-base crosslinking to reduce the initial burst release of methotrexate (MTX) by 58.4% under the experimental conditions reported in the original study [39]. To realize multifunctional integration, the incorporation of supramolecular chemistry into carrier design has shown substantial promise [40]. Wang et al. developed a dual-dynamic cross-linked hydrogel, designated SPT@TPL, based on sodium alginate. This hydrogel exhibits not only ROS-responsive and self-healing properties but also enables the controlled release of triptolide (TPL) for up to 30 days within the high-ROS microenvironment of RA, thereby significantly mitigating inflammation and promoting articular cartilage repair [41]. Zhou et al. constructed a β-cyclodextrin/adamantane supramolecular hydrogel (DGHCAP) that synergizes graphene oxide (GO) π-π stacking with host–guest interactions, achieving a reported caffeic acid loading efficiency of 43.38% [42]. In the corresponding experimental study, the system was also associated with reductions of approximately 65% in IL-6 and 72% in TNF-α, together with sustained drug release and antioxidant activity.

While hydrogels demonstrate distinct advantages in advanced drug delivery systems, their inherent mechanical deficiency remains a critical translational constraint. Contemporary researchers are addressing this challenge through innovative material engineering approaches. Chen’s team implemented a plasma-enhanced surface engineering protocol on alginate-tyramine hydrogels (ALG-TYR), achieving radical-mediated amide crosslinking within 5 min that elevated compressive strength to 200 kPa—matching native cartilage biomechanics—while eliminating exogenous crosslinker-associated biocompatibility risks [43]. Collectively, hydrogel systems establish an innovative paradigm for precision drug delivery through tunable physicochemical properties and multi-scale spatiotemporal regulation. The future development needs to focus on multi-drug synergistic delivery, intelligent response system and cross-scale theoretical modeling, combined with gene editing and bioelectronic technology to promote personalized medicine [44]. Hydrogels will continue to lead biomedical material innovation with their potential to enhance therapeutic efficacy and reduce toxic side effects. Taken together, the representative hydrogel studies illustrate that their therapeutic value is strongly dependent on the combination of cargo properties, administration route, cross-linking strategy, and pathological target. Intra-articular hydrogels are mainly investigated as local depots for small-molecule drugs, nucleic acids, or biologically active agents, with sustained retention and controlled release serving as the major design objectives. However, prolonged release alone does not necessarily translate into improved therapeutic outcomes, and the effects of each formulation should be evaluated together with the disease model, inflammatory or structural endpoints, and safety profile. In addition, the incorporation of nanozymes, graphene oxide, or other functional components may improve specific therapeutic functions but also increases formulation complexity and may introduce additional safety and manufacturing concerns. Therefore, hydrogel systems should be compared not only by release duration or mechanical properties but also by therapeutic benefit, local retention, safety, and translational feasibility.

### 5.2. Polysaccharide-Based Nanoparticles

The application of nanoparticles in RA drug delivery has been extensively studied. Nanoparticles—colloidal particles typically under 1000 nm—leverage polysaccharide-based designs to enhance insoluble drug solubility, improve tissue targeting, and achieve controlled drug release [45]. Owing to the facile functional modification of polysaccharides, most research focuses on modifying polysaccharides to further optimize nanoparticle delivery efficacy.

Common polysaccharide nanoparticle synthesis methods include cross-linking, emulsification, and ion-gelation [46]. This section describes these prevalent synthetic approaches alongside research on polysaccharide-derived nanoparticles (Table 1).

**Figure 4:**
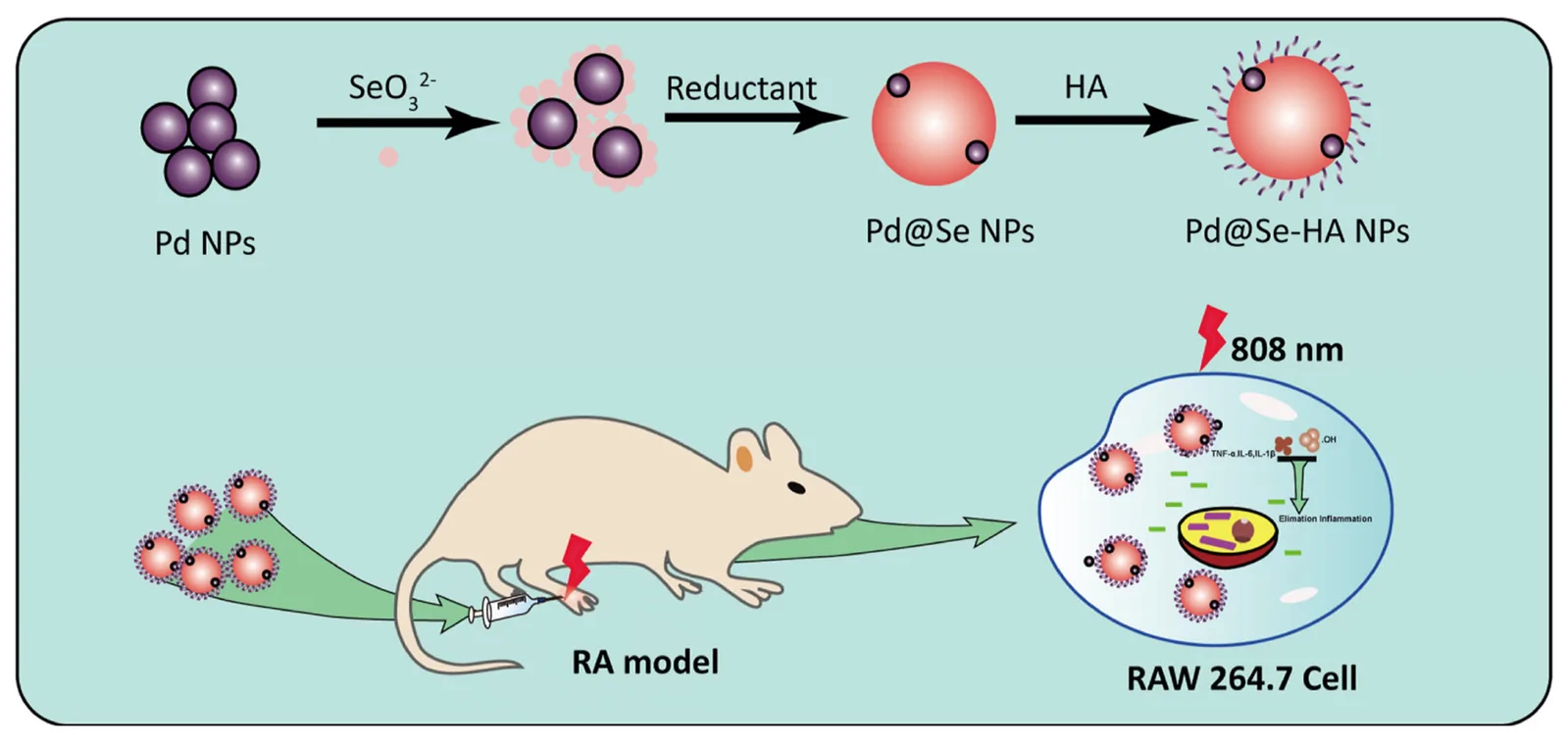
The process of palladium (Pd) and HA integrated into selenium (Se) nanoparticles (Pd@Se-HA NPs) synthesis and therapy RA by combination therapy inhibiting the macrophage inflammatory response in vivo. Arrows indicate the nanoparticle synthesis and treatment sequence, while the colors and symbols represent the corresponding nanoparticles, RA model, and RAW 264.7 cells as depicted. Reproduced from Ref. [49] under the Creative Commons Attribution-NonCommercial-NoDerivatives 4.0 International License.

Drug loading in nanoparticles is typically achieved by encapsulating therapeutics within their matrix or adsorbing them onto the surface through methods such as physical entrapment or electrostatic adsorption. Current research on polysaccharide-derived nanoparticles prioritizes expanding drug-loading capacity, achieving multi-stimuli responsiveness, and enhancing targeting precision [47]. This drives our commitment to developing multifunctional polysaccharide-based nanocarriers. The photothermal effect—wherein materials convert light energy into localized heat—has been innovatively applied to drug delivery [48]. Zheng et al. designed HA-modified core–shell Pd@Se nanoparticles (Pd@Se-HA NPs; shown schematically) [49]. Employing electrostatic adsorption-reduction, they integrated a photothermal Pd core with a radical-scavenging Se shell. HA–CD44-mediated receptor recognition and macrophage uptake, together with the intrinsic photothermal and radical-scavenging properties of the nanoparticles, was associated with 98.6% •OH-scavenging activity and a reduction in the reported inflammation score from 4.0 to 0.5 over 15 days under the conditions of the original study (Figure 4). Similarly, Yang et al. developed biomimetic CS-chondroitin sulfate nanoparticles (CS-CHI NPs) by grafting chondroitin sulfate onto CS nanoparticles via EDC/NHS coupling, replicating natural glycan “bottle-brush” topology [50]. Subsequent physical adsorption of DS into the crosslinked network addressed the critical coexistence of lubrication deficiency and inflammation in arthritis therapy. Receptor recognition and effective in vivo targeting should be distinguished when evaluating targeted delivery systems. HA–CD44 interactions may facilitate receptor recognition and cellular uptake, particularly in CD44-expressing cells, but receptor binding alone does not demonstrate preferential accumulation in inflamed joints or improved therapeutic targeting. Stronger evidence for in vivo targeting would include quantitative biodistribution, tissue drug concentration, cellular localization, pharmacokinetic analysis, or receptor-blocking studies. Therefore, in this review, receptor-mediated recognition is distinguished from tissue accumulation and therapeutic targeting when interpreting the available evidence.

Stimuli-responsiveness constitutes a core design strategy for next-generation smart drug delivery systems. In RA, several pathological features of the inflamed joint can provide endogenous cues for stimulus-responsive drug release, including local acidosis, oxidative stress, and elevated proteolytic activity. However, these signals should not be regarded as fixed or disease-specific thresholds because their magnitude varies among patients, disease states, anatomical sites, and analytical methods. For example, reported synovial fluid pH values in RA have reached approximately 6.8–7.1, compared with approximately 7.4–7.8 in healthy individuals, although substantial inter-study and inter-individual variation has been reported depending on sampling and measurement conditions [51,52]. Thus, pH-responsive systems should be designed to respond to a pathophysiologically relevant acidic shift rather than assuming a universal RA-specific pH threshold.

Proteolytic activity provides another clinically relevant trigger. In a clinical study, synovial fluid MMP-9 concentrations were significantly higher in patients with RA than in patients with osteoarthritis (16.17 ± 12.25 vs. 0.75 ± 0.53 ng/mL, *p* < 0.001), and MMP-9 levels were also higher in erosive than in non-erosive RA (18.43 ± 12.87 vs. 9.36 ± 7.72 ng/mL, *p* < 0.05) [53]. These findings support the pathological relevance of MMP-9 as an enzymatic cue for responsive delivery, while also indicating that MMP-9 abundance varies with disease phenotype. Accordingly, MMP-responsive systems should be interpreted as exploiting elevated proteolytic activity within inflamed joints rather than relying on a universal concentration threshold.

Oxidative stress is another important feature of the inflammatory RA microenvironment and has been exploited in ROS-responsive delivery systems. Unlike pH or individual proteases, however, there is currently no universally accepted quantitative ROS concentration that can be used as a standardized threshold for the RA joint microenvironment. ROS measurements are strongly dependent on the reactive species examined, biological compartment, detection probe, and analytical method. Therefore, ROS-responsive systems are more appropriately considered to respond to a relative increase in local oxidative stress rather than to a fixed RA-specific ROS concentration. This distinction is important when evaluating the translational relevance of stimulus-responsive systems and when comparing results obtained using different experimental models. As exemplified by the Abies polysaccharide (Abps) system, researchers chemically conjugated curcumin (Cur) and tretinoin (Cel) via a ROS-responsive thioredoxin bond and an enzyme-sensitive polypeptide (PVGLIG), forming dual-responsive hybrid micelles (D&A@Cel) [54]. This system utilizes dextran sulfate (DS) to target macrophage scavenger receptors (SR-A), while achieving drug release through dual triggering by joint microenvironmental matrix metalloproteinases (MMP-2/9) and ROS. Animal studies demonstrated enhanced drug bioavailability and anti-inflammatory efficacy (particle size: 204.8 nm; 40% increase in joint swelling inhibition rate). In the layered double hydroxide (LDH) system, MTX was intercalated into LDH via ion exchange, with DS modification enabling active targeting (particle size: 303.1 nm; zeta potential: −12.4 mV) [55]. This nanocomplex (LDH-MTX-DS) exhibited pH-sensitive release, with 79.3% cumulative drug release over 72 h under the acidic experimental condition (pH 5.5), compared with 66.9% at physiological pH. Notably, these experimental pH values represent in vitro release conditions rather than direct measurements of RA synovial fluid and therefore should not be interpreted as defining a universal RA microenvironmental threshold.

**Table 1:** Common synthesis methods for polysaccharide nanoparticles.

Synthesis Method	Principle	Applicable Polysaccharide Types	Key Advantages	Representative RA Application	Reference
Crosslinking	3D network structure through covalent or ionic bonding	CS, sodium alginate, HA	High stability, pH responsive release (ionic crosslinking)	Injectable hydrogels or nanoparticles for sustained intra-articular delivery	[56]
Self-assembly method	Spontaneous formation of micelles/vesicles in water by amphiphilic polysaccharides	Hydrophobic polysaccharides (e.g., cholesterol-CS, acetylated dextran)	High drug loading (>20%), pH/enzyme responsive release	Polysaccharide-based micelles/vesicles for hydrophobic drug or co-drug delivery	[57,58]
Nanoprecipitation	Add drops of polysaccharide solution to a poor solvent to induce precipitation and form nanoparticles.	Cellulose derivatives, starch	Simple and fast operation without high temperatures	Polysaccharide nanoparticles for controlled delivery of anti-inflammatory agents	[59,60]
Emulsification	Water/oil phase emulsification followed by volatile solvent curing	Sodium alginate, pullulan polysaccharide	Suitable for hydrophobic drugs with controlled particle size (100–500 nm)	Polysaccharide microspheres/nanoparticles for sustained or controlled drug delivery	[61]
Ionic gelation	Gelation of ionic polysaccharides with counterions by electrostatic interaction	CS (+) with TPP (−), sodium alginate (−) with Ca^2+^	Gentle conditions, rapid prototyping (minute level)	Chitosan/alginate-based nanoparticles for localized or sustained delivery of anti-inflammatory drugs	[62]

### 5.3. Polysaccharide-Based Microneedles

Microneedles (MNs) are minimally invasive transdermal technologies that bypass the stratum corneum via micrometer-sized protrusions (typically <2000 μm), enabling efficient therapeutic delivery [63]. Compared to hydrogels and nanoparticles, microneedles eliminate repeated injections and permit patient self-administration. Polysaccharide derivative microneedles retain inherent properties from their constituent polysaccharides and exhibit distinctive advantages over other MN types, e.g., biocompatibility and tunable solubility [64]. Certain polysaccharides possess inherent anti-inflammatory effects—particularly valuable for RA—enabling dual therapeutic action when designed as microneedle matrices. For example, chondroitin sulfate C (CSC), the predominant articular cartilage glycosaminoglycan with demonstrated anti-inflammatory properties, is suitable for microneedle fabrication [65]. Bui et al. designed dissolvable CSC-based microneedles (EV@cMN) loaded with human adipose stem cell-derived extracellular vesicles (hASC-EVs) at needle tips using vacuum filling and layered drying [66]. The hASC-EVs were delivered through the skin by the dissolvable CSC microneedles, and the reported therapeutic effects in CIA models were associated with modulation of M1 macrophage polarization and inhibition of activated synovial fibroblast migration. Sustained release of CSC further contributed to the anti-inflammatory effects and cartilage regeneration observed in the model. In collagen-induced arthritis (CIA) models, EV@cMN treatment was reported to reduce joint swelling by 70% and histological scores for synovitis, bone erosion, and cartilage destruction by more than 60% under the experimental conditions of the study, with no obvious systemic toxicity reported.

Jin’s team similarly utilized chondroitin sulfate (CS), employing negatively charged CS and positively charged bee venom peptide (MEL) to neutralize MEL’s membrane-disrupting toxicity through electrostatic complexation (MC complexes) [67]. These complexes were subsequently loaded into cryo-microneedle patches (MC@CCMNPs) constructed from methacrylated chondroitin sulfate (CSMA) for localized sustained delivery. This strategy synergistically mitigated MEL acute toxicity while enhancing therapeutic safety. Similarly, Liu et al.’s fucoidan (Fuc) microneedle system achieved synergistic macrophage M1/M2 polarization modulation by loading anti-inflammatory drugs via ROS-responsive polymers (FTL@SIN) [68]. Beyond leveraging intrinsic polysaccharide activity, integrating microneedle technology with sensing-control systems enables smarter precision delivery, particularly for therapies requiring strict blood drug monitoring. For example, Parrilla et al. designed a wearable closed-loop system using hollow microneedle arrays [69]. Featuring chitosan-coated electrochemical sensors and iontophoretic delivery, it addresses delayed monitoring and inefficient transdermal delivery in MTX therapy. The sensor demonstrated biofouling resistance and >2-day stability, while combined ion electroosmosis achieved on-demand release (26-fold ex vivo efficiency gain), offering novel closed-loop management for cancer, RA and other diseases.

Overall, polysaccharide-based microneedles provide a minimally invasive route for transdermal delivery and may reduce the need for frequent parenteral administration in selected settings. However, current evidence does not establish direct delivery of microneedle cargos to the synovial cavity. Their therapeutic effects may involve systemic absorption, lymphatic transport, local tissue distribution, or a combination of these pathways, depending on microneedle design and cargo properties. Compared with intra-articular delivery, microneedles offer advantages in administration convenience and invasiveness but may provide less direct control over intra-articular drug exposure. Further studies comparing pharmacokinetics, biodistribution, synovial drug concentration, and therapeutic efficacy with intra-articular administration are therefore needed. Future integration with 3D printing technology could enhance manufacturing precision and efficiency, further advancing their applications in drug delivery [70].

### 5.4. Other Carrier Forms

Hydrogels, nanoparticles, and microneedles—as core carriers in polysaccharide-derived drug delivery systems—offer distinct advantages for RA treatment through their unique physicochemical properties and targeting mechanisms. Concurrently, novel polysaccharide-based vehicles (e.g., microspheres, vesicles, and microsponges) emerge as research focuses enabled by molecular modification advances. With differentiated drug-loading modes and release kinetics—including microspheres’ sustained-release properties, vesicles’ membrane functionalization capacity, and microsponges’ high porosity—these systems provide complementary solutions to key RA therapeutic challenges: prolonged intra-articular drug residency, precise synovial/macrophage targeting, and synergistic multidrug delivery.

Polymeric vesicles (PVs) are self-assembled hollow spherical structures based on amphiphilic block copolymers. They encapsulate both hydrophilic/hydrophobic drugs while enabling stimulus-responsive behavior through polymer design [71]. Compared to lipid vesicles, polymer vesicles exhibit enhanced stability and functional versatility. As drug carriers, vesicles require optimization of biocompatibility, drug loading efficiency, and membrane properties [72]. Polysaccharide-derived vesicles, as a typical representative of polymer vesicles, exhibit superior biocompatibility, active targeting and microenvironmental responsiveness in the treatment of RA. For example, HA, a natural glycosaminoglycan, is biocompatible and specifically binds CD44 receptors overexpressed on synovial inflammatory cells. Leveraging HA’s properties, Wang et al. designed stimuli-responsive hyaluronic acid-triglycerol monostearate dual-drug vesicles (HTDD) for co-delivering (2-deoxy-D-glucose) 2-DG and dexamethasone (Dex) to arthritic sites [73]. HA and triglycerol monostearate (TGMS) were conjugated via acid-labile 3-aminophenylboronic acid to form an amphiphilic copolymer. The hydrophobic anti-inflammatory drug Dex formed a film with the copolymer, encapsulating hydrophilic glycolysis inhibitor 2-DG upon hydration. This design enables 2-DG to inhibit synoviocyte glycolysis and prevent Dex efflux, while Dex suppresses inflammatory signaling and mitigates 2-DG-induced oxidative stress. The vesicles respond to MMP overexpression (enzymatically cleaving TGMS chains) and acidic pH (hydrolyzing borate bonds) in RA synovium, triggering targeted drug release. Hu’s team also utilized HA, innovatively modifying HA with Fmoc-phenylalanine dipeptide (FmocFF) to construct nano-vesicles (HA-FP) exhibiting hierarchical self-assembly [74]. The self-assembly of HA molecular chains into vesicles was driven by FmocFF β-folding, with curcumin (Cur) incorporated into vesicle walls via π-π interactions. This design preserves HA’s lubrication function and CD44 targeting while significantly enhancing enzymatic degradation resistance (6-fold molecular weight retention after 6 h hyaluronidase exposure), moreover achieving anti-inflammatory efficacy through down-regulating macrophage TNF-α/IL-1β secretion and inducing apoptosis of activated cells. Animal studies demonstrated marked reduction in joint edema and significant repair of cartilage pathological damage.

**Figure 5:**
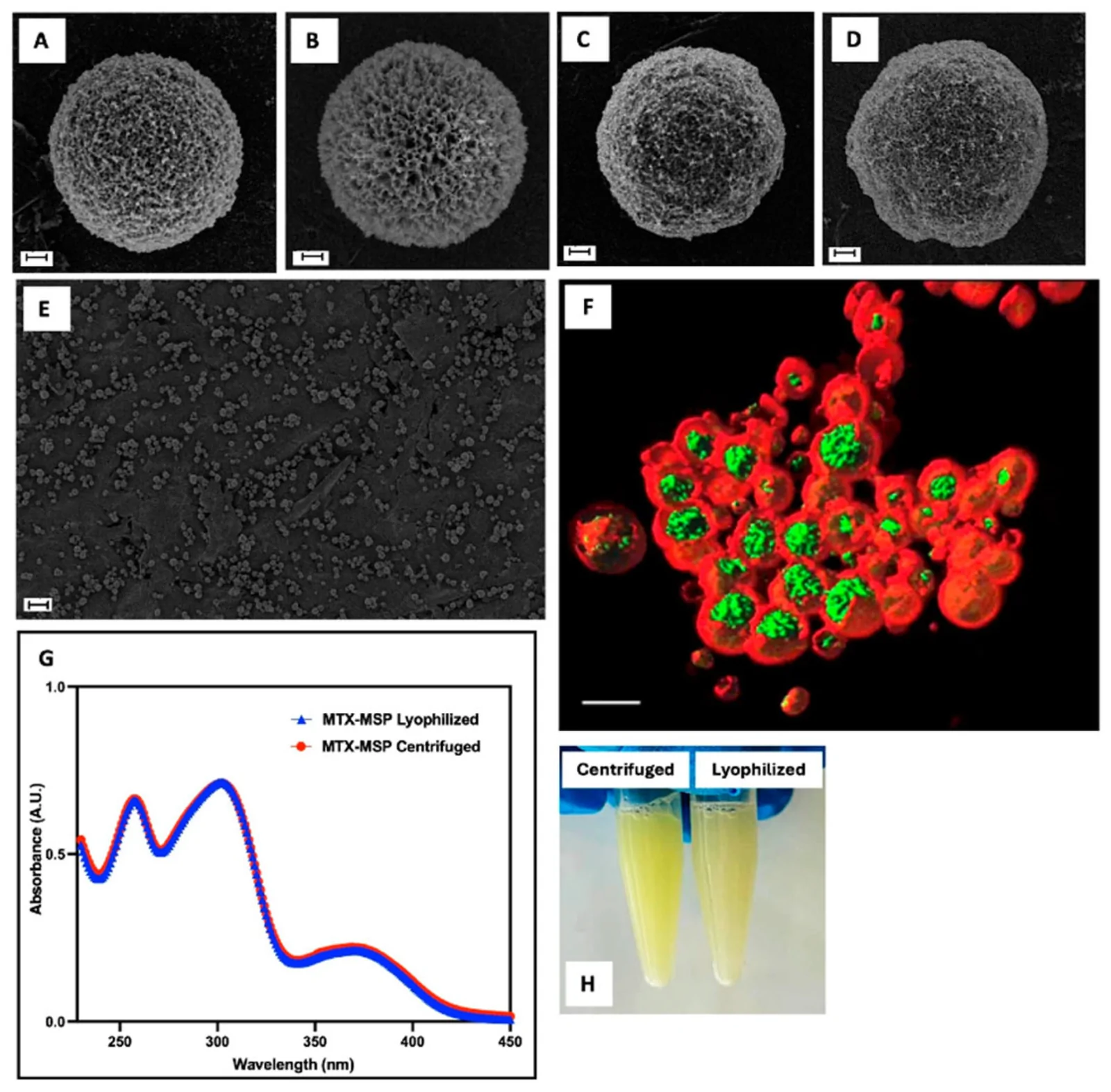
SEM images of the MSP before the freeze-dry step (**A**) and after the freeze-dry step (**B**) and MSP loaded with MTX after centrifugation (**C**) and after freeze-drying (**D**) (scale bar: 1 μm). Morphology of the MTX-MSP population characterized by SEM (**E**) (scale bar: 20 μm) and confocal analysis (**F**) (3D rendering with isosurfaces; green indicates MTX, red indicates MSP; scale bar: 5 μm). MTX spectra of MTX-MSP obtained by UV-Vis following centrifugation and lyophilization processes (**G**). Photograph of MTX-loaded MSP after centrifugation, lyophilization, and reconstitution in PBS buffer (**H**). Reproduced from Ref. [76] under the Creative Commons Attribution 4.0 International License.

Microsponges (MSPs) are spherical aggregates typically ranging from 5 to 300 μm in size, characterized by structured microcavities formed through continuous nanochannel networks. Current MSP systems face limitations in industrial production due to their complex preparation processes and insufficient long-term storage stability. However, exploiting their structural features, MSPs offer significant drug delivery advantages, including high loading efficiency and controlled drug release [75]. Combining these attributes with polysaccharides’ inherent benefits, polysaccharide-derived MSP demonstrate natural biocompatibility, biodegradability, and microenvironmental responsiveness for RA treatment, emerging as innovative carriers that overcome traditional delivery limitations. Fiaschini et al. developed HA MSP employing disulfide bond dynamic cross-linking to construct interconnected nanopores, with lyophilized powder formulations enabling efficient MTX loading and stable storage [76]. This design leverages HA’s natural CD44 targeting to guide 20–60 μm carriers toward inflamed synovial membranes while implementing redox responsiveness through disulfide bond cleavage in high-ROS microenvironments (Figure 5). Daniela Ariaudo’s team designed alginate MSP for peptide drug (CIGB814) delivery suitable for oral administration [77]. Their innovative gas-foaming cross-linking approach spontaneously generates 50–120 nm through-pores via in situ CO_2_ production from reactions between disulfide cross-linker (DIDS) and alginate carboxyl groups. Compared with physical embedding, this approach was reported to preserve peptide conformational integrity through surface-adsorption-dominated loading, with a reported loading efficiency of 75%. Thus, polysaccharide-derived MSP represent promising drug delivery carriers that may advance RA therapeutic strategies.

Polymer microspheres represent spherical polymer carriers that enable controlled drug release through leaching or degradation mechanisms [78]. These carriers are available in sizes ranging from 1 to 250 μm, depending on specific requirements and preparation methodologies. They exhibit significant advantages for drug delivery applications, including high design flexibility, facile surface modification capabilities, substantial specific surface area, and enhanced structural stability [79]. Leveraging the inherent advantages of polysaccharides as polymeric microsphere materials, polysaccharide derivative microspheres hold significant potential for application in RA therapy. Among therapeutic agents for RA, meloxicam (MLX), a non-steroidal anti-inflammatory drug, is hampered by poor water solubility, gastrointestinal irritation, and a short half-life. Abadi et al. designed colon-targeted adhesive MLX microspheres. Utilizing the gelation capability of sodium alginate and the mucosal adhesive properties of aloe vera gel powder (AGP), the researchers employed an ionic gelation method [80]. They used CaCl_2_ as a cross-linking agent to cross-link the sodium alginate, thereby enhancing structural stability. Within this design strategy, AGP serves to increase MLX solubility and prolong colonic retention time. This effect arises because AGP can bind to intestinal mucosal mucin through interactions with the carboxyl/hydroxyl groups of sodium alginate. Although research on polysaccharide derivative microspheres remains at a developing stage, it represents a novel and promising approach for RA therapy [76].

In summary, novel delivery vehicles such as vesicles, microsponges, and microspheres constructed from polysaccharide derivatives expand the functional repertoire of polysaccharide DDS and address specific unmet needs (e.g., oral delivery, dual-drug co-loading). However, these emerging systems are currently at an early proof-of-concept stage: most studies only report preliminary efficacy in acute animal models, with scarce data on long-term biocompatibility, repeat administration safety, and scale-up feasibility. In particular, the complex preparation processes of vesicles and microsponges pose significant challenges for industrial production and quality control. While further optimization regarding scale-up production and long-term stability is required, these carriers’ demonstrated superior performance provides a robust foundation for developing more efficient and safer RA-targeted therapeutic strategies [81,82].

### 5.5. Composite Delivery Systems

In previous sections, fundamental polysaccharide-based drug delivery carriers were summarized. Within the drug delivery field, research on composites integrating multiple fundamental carriers mentioned above has also garnered significant interest. Based on matrix composition, polysaccharide-containing composite drug delivery systems can be classified into two categories: the first comprises entirely polysaccharide-based primary delivery carriers that are intercombined; the second is formed by combining polysaccharide-based carriers with non-polysaccharide-based carriers, such as those derived from synthetic polymers. According to carrier type, common composite delivery systems include nanoparticle-hydrogel, nanoparticle-microneedle [83], and microsphere-hydrogel systems. Although compositing may introduce new challenges (e.g., nanocomponent biocompatibility risks, complexity of multiphase interfacial interactions), it also enables synthesis of different vectors’ strengths, offering significant potential to overcome functional limitations of single-vector systems. This section describes different polysaccharide composite drug delivery carriers for RA treatment based on carrier type.

The nanoparticle-hydrogel composite represents the most common polysaccharide composite delivery system. The hydrogel structure directly encapsulates nanoparticles with good interfacial stability through hydrogen/coordination bonds. Weng et al. designed a triple-synergistic nanoparticle-hydrogel composite using alginate (ALG) hydrogel [84]. Positively charged poly (pyrrole) (Ppy) NPs and polyethyleneimine (PEI)-modified MoS_2_ NFs were attracted via electrostatic binding to negatively charged ALG chains for hydrophilic strontium ranelate (SrR) delivery. In this design, the NFs serve as photomagnetized materials while Ppy NPs act as photothermal conversion materials. The synergistic integration of photothermal therapy and photoresponsive nanomaterials yielded a composite hydrogel system with functions including drug retardation, skin penetration enhancement, and immunomodulation [85].

Siddiqui et al. designed a folic acid-modified chitosan-chondroitin sulfate nanoparticle-loaded hydrogel [86]. Utilizing ionic cross-linking, they prepared targeted nanoparticles (FA-CHI bound to CHS) and a transdermal hydrogel delivery system containing almond oil as a pro-osmotic agent. This system resolved leflunomide’s (LEF) issues of low oral bioavailability, high systemic toxicity, and inadequate RA targeting. It addressed LEF’s low oral bioavailability, high systemic toxicity, and insufficient RA-targeted therapy by demonstrating pH-responsive drug release, with a reported cumulative release of 91% under the inflammatory-site conditions used in the original study, together with macrophage targeting, enhanced transdermal penetration, and reduced systemic toxicity. Consequently, nanoparticle-hydrogel carriers hold significant promise in composite delivery systems and will inspire development of other composite delivery platforms.

Nanoparticle-microneedle composite carriers consist of nanoparticles encapsulated within microneedles, combining the nanoparticle advantages of drug activity protection and solubility enhancement with the microneedle benefits of painless transdermal delivery and self-administration [87,88]. To address the high toxicity and low efficiency of existing MTX monotherapy microneedles for RA, Wang et al. designed a combined delivery system (GA-CDs@MTX MNs) using glycyrrhetinic acid (GA)-modified carbon-dot nanocomplexes (CDs) within HA microneedles [89]. The CDs were synthesized from Poria cocos polysaccharides via one-step hydrothermal processing, leveraging surface-exposed active-NH_2_ groups. GA modification onto CDs was achieved through amide reaction utilizing GA’s -COOH groups, with subsequent MTX loading onto GA-CDs forming GA-CDs@MTX nanoparticles for incorporation into HA microneedles. This design strategy utilized CDs’ photoluminescence for fluorescence monitoring while combining GA’s inherent anti-inflammatory effect with MTX for dual anti-inflammation. Liu et al. developed a nano-micro-needle system (FTL@SIN MNs) employing fucoidan (Fuc) as an anti-inflammatory hydrophilic backbone [68]. ROS-responsive thioketone (TK) linkages conjugated lignocaine-loaded cycloheximide (SIN) to Fuc, forming FTL@SIN nanoparticles loaded into fucoidan-based microneedles. This yielded ROS-sensitive dual anti-inflammatory composite carriers. The microneedle preparation featured innovative design: Fuc provided enhanced needle-tip integrity and mechanical strength compared to HA, while HA was strategically used for the needle backing to improve flexibility. Nanoparticle-microneedle composite carriers possess unique advantages warranting increased future attention.

Distinct from nanoparticle-hydrogel composite carriers—where substantial specific surface area enables rapid drug loading and high surface loading rates—microsphere-hydrogel composite carriers achieve smoother, more sustained drug release profiles. Here, microspheres serving as primary drug reservoirs are entrapped within the hydrogel system. This hydrogel functions as a physical barrier, utilizing its adjustable cross-linking degree lattice structure to restrict drug diffusion, thereby facilitating sustained release [90]. These systems have also attracted significant attention. Zhang et al. developed an injectable OHA/QCS hydrogel encapsulating diacerein-loaded PLGA microspheres (DIA-MS@gel) for intra-articular delivery in osteoarthritis (OA) [91]. The hydrogel matrix was formed through dynamic crosslinking between aldehyde groups of OHA and amino groups of QCS, providing a pH-responsive network that regulated microsphere degradation and drug release. The composite prolonged in vitro drug release for up to 47 days and maintained drug release in the rabbit joint cavity for more than 31 days after intra-articular administration, while also promoting cartilage repair in an OA model. These findings demonstrate the potential of polysaccharide-containing microsphere–hydrogel composites for prolonging intra-articular drug exposure. However, because this study was conducted in OA rather than RA, its therapeutic outcomes should not be interpreted as direct evidence of efficacy in RA. In an earlier study, Chen et al. developed a brucine-loaded chitosan microsphere/thermoresponsive chitosan–glycerol–borax hydrogel system for intra-articular administration [92]. Incorporation of the microspheres into the thermoresponsive hydrogel substantially retarded the burst release observed from the microspheres alone, and the formulation remained in the joint cavity for more than 7 days in rats. The study primarily evaluated the system in an OA model, although the authors discussed its broader potential for rheumatic diseases. Thus, this work provides additional evidence supporting the general strategy of combining microspheres with hydrogels to improve intra-articular retention, but it should not be regarded as direct validation of a polysaccharide-derived DDS for RA.

Wang et al. developed a diphenyl carbonate crosslinked β-cyclodextrin nanosponge-based topical gel for co-delivery of baricitinib and tenoxicam in RA [93]. The system realized high drug loading, sustained biphasic release, favorable skin biocompatibility, and synergistic anti-arthritic efficacy via JAK/COX dual-pathway inhibition, showing good potential in joint protection and systemic toxicity reduction. Nevertheless, the nanosponge composite suffered from relatively large particle size, lack of inflammatory microenvironment responsiveness, and insufficient active targeting. In addition, the study was limited to preliminary animal evaluation without data on skin pharmacokinetics, long-term stability, and clinical translation. Therefore, although cyclodextrin nanosponge–hydrogel composites provide a feasible strategy for topical RA treatment, further optimization is still required in intelligent responsiveness, precise targeting, and clinical practicability.

In summary, polysaccharide-derived composite DDS can compensate for the inherent shortcomings of single carriers and achieve synergistic functional upgrades. However, increasing structural complexity inevitably raises manufacturing difficulty, reduces batch-to-batch reproducibility, and increases production costs—all of which are unfavorable for clinical translation [94]. In addition, the in vivo fate of composite systems remains poorly understood, and the organ accumulation risk of nanoscale components requires more rigorous investigation. Overall, composite systems represent an important developmental direction, but design priorities should focus on solving clear clinical problems rather than pursuing excessive structural complexity. Importantly, additional structural components should provide a measurable therapeutic or functional benefit that justifies the associated increase in synthetic steps, characterization requirements, regulatory burden, and safety risks. In the absence of such comparative evidence, increasingly complex multifunctional architectures should not be assumed to provide superior overall therapeutic value.

## 6. Polysaccharide Derivative Carrier Design

Natural polysaccharide materials possess inherent biomedical advantages, underpinning the importance of their design and application. This chapter will discuss design considerations for natural polysaccharide derivative carriers and systematically classify different carrier types.

### 6.1. Polysaccharide Design Directions

To harness the unique advantages of polysaccharides while addressing their inherent limitations, the design of polysaccharide-based drug delivery carriers typically follows two strategic pathways. Firstly, polysaccharides can serve as the core material, leveraging their naturally abundant functional groups for chemical modification to introduce functionalities such as targeting and stimulus responsiveness. This approach helps retain intrinsic properties like biocompatibility and biodegradability. Alternatively, polysaccharides may be incorporated as functional modules into other polymeric systems, enhancing key properties of the host material—including hydrophilicity/hydrophobicity, biocompatibility, and targeting ability [95].

The overarching goal in designing such carriers is to balance innate polysaccharide characteristics with performance demands: preserving advantages such as biosafety, abundant sourcing, and environmental sustainability, while overcoming drawbacks including limited mechanical strength, solubility, and stability through molecular modification. Expanded functions such as targeting and responsiveness are also incorporated. For example, although the inherent hydrophilicity of polysaccharides facilitates drug loading, excessive water solubility may reduce loading efficiency, often necessitating the introduction of hydrophobic groups or composite material strategies to achieve an optimal balance. Moreover, the degradation profile of polysaccharides must be carefully aligned with drug release kinetics to avoid issues associated with premature breakdown or undesired persistence [26,27].

For example, grafting heparin onto polymer surfaces confers anticoagulant properties; modifying HA onto nanoparticles enables CD44 receptor-mediated tumor targeting [96]; and attaching β-glucan fragments with immunostimulant activity to carriers enhances adjuvant effects [97]. This strategy greatly expands the flexibility of vector design, enabling the “endowment” of polysaccharides with excellent biological properties to high-performance synthetic materials that do not otherwise possess such properties, creating “hybridized” multifunctional vectors. The abundance of reactive groups (hydroxyl, amino, carboxyl) on polysaccharides makes it easy to efficiently graft them through a variety of chemical reactions (e.g., amidation, esterification) [30]. In conclusion, polysaccharide materials can be easily modified or grafted onto other polymers because they mainly contain a large number of hydroxyl, amino and carboxyl groups. Selection of suitable polysaccharides and reactions can reduce the cost and improve the preparation efficiency.

### 6.2. Stimulus Response Design

Owing to the toxicity and side effects of conventional RA drugs, achieving precise spatiotemporal drug release is crucial for effective treatment. Stimuli-responsive drug carriers respond to chemical/physical signals from internal or external environments, such as ROS levels, pH, temperature, and magnetic fields. Incorporating stimuli responsiveness has significantly enhanced drug release precision in traditional carriers. These responses are broadly categorized as internal or external stimuli responsiveness [98,99]. Polysaccharide-based delivery systems possess distinct advantages for developing such responsiveness: Certain polysaccharides intrinsically respond to specific physicochemical properties or biological signals, while their abundant surface functional groups (e.g., hydroxyl, amino) facilitate grafting of stimulus-responsive molecules. Thus, polysaccharide derivatives represent an ideal platform for constructing intelligent responsive carriers [100].

RA inflammation induces lactic acid accumulation within the synovium, generating a weakly acidic joint microenvironment distinct from normal tissues. Leveraging this distinct pathological feature, drug delivery carriers can be endowed with pH-responsive properties, enabling drug release at low-pH inflammatory sites. This approach achieves precise drug delivery while increasing therapeutic concentration within inflamed joints. Generally, pH-responsiveness is conferred through two strategies: The first introduces acid-labile bonds (e.g., hydrazone [101], imine, or ketal bonds) into carriers [102,103]. The cleavage behavior of these acid-labile linkages is strongly dependent on their molecular structure and surrounding chemical environment and should not be inferred solely from the relatively acidic conditions of the RA joint. Therefore, pH-responsive carriers should be experimentally validated under physiologically relevant RA conditions rather than assuming efficient cleavage solely because the joint microenvironment is relatively acidic. The second strategy utilizes polysaccharides or modified derivatives containing weak acid/base groups. Protonation/deprotonation in acidic environments alters surface charge distribution and hydrophilic-hydrophobic balance, subsequently enhancing polysaccharide derivative swelling or inducing polymer aggregation.

Abnormal expression of multiple enzymes in RA joints—including hyaluronidase and matrix metalloproteinase MMP-2—participates in inflammation and joint destruction. Leveraging RA-specifically expressed enzymes enables the design of enzyme-responsive polysaccharide derivatives as carrier substrates. These systems utilize polysaccharides degradable by specific enzymes (e.g., HA by hyaluronidase). At enzyme-overexpressed inflammatory sites, accelerated carrier degradation releases drugs. Carrier resistance to enzymatic degradation can also be enhanced through modification to prolong circulation time. Ali et al. constructed an enzyme-responsive amphiphilic polymer SA-PL by grafting hydrophobic drug SA onto hydrophilic polymer PL via ester bonds [104]. This polymer self-assembles into nanomicelles (SA-PL NMs) in aqueous media, subsequently encapsulating drug Reb to form Reb@SA-PL NMs. The enzyme-responsive mechanism leverages overexpression of MMP-13 and esterase in arthritic synovial fluid to cleave micellar ester bonds. Beyond this approach, other RA-specifically expressed enzymes offer opportunities for responsive carrier design.

Stimulus-responsive design constitutes the core precision strategy for polysaccharide drug carriers. By leveraging RA joint pathology (reduced pH, ROS bursts, specific enzyme overexpression), pH-responsive, ROS-responsive, enzyme-responsive, and multi-responsive mechanisms enable precise spatiotemporal drug release at target sites, significantly enhancing therapeutic efficacy while minimizing adverse effects. That said, the actual clinical value of multi-responsive design requires prudent evaluation: the magnitude of pathological stimuli varies greatly among patients and disease stages, which may lead to inconsistent drug release behavior, and non-specific cleavage of responsive bonds by off-target biomolecules can cause premature drug release in vivo. For clinical translation, prioritizing one dominant, robust responsive mechanism rather than pursuing an excessive number of response types may represent a more pragmatic strategy [105].

### 6.3. Drug Loading Methods

The selection of an appropriate drug loading method for different RA therapeutic agents and drug delivery carriers is critical to enhance drug utilization efficiency and optimize treatment outcomes. When choosing a drug loading strategy, it is essential to take into comprehensive consideration the hydrophilicity/hydrophobicity properties of the drug, as well as the characteristics of polysaccharide derivative-based delivery systems. Commonly employed drug loading techniques include physical entrapment and chemical grafting, among others [35].

Physical entrapment relies on non-covalent interactions, such as hydrogen bonding and hydrophobic effects, to incorporate drugs. The primary advantage of this approach lies in its operational simplicity, as it avoids chemical reactions and thus preserves the structural and physicochemical integrity of the carrier, rendering it especially suitable for small-molecule drugs. Currently, the majority of polysaccharide-based delivery systems utilize physical entrapment for drug incorporation [106]. For instance, in polysaccharide-based hydrogel systems, drugs can be loaded into the three-dimensional network via physical mixing [107,108]. Zhou et al. developed a supramolecular hydrogel (DGHCAP) composed of graphene oxide (GO)-modified HA and adamantane (AD)-modified polyethylene glycol (PEG), which was physically incorporated with cichoric acid (CA) [42]. Although based on physical mixing, the benzene ring of CA can engage in π–π stacking interactions with the aromatic domains and oxygen-containing functional groups (e.g., –OH, –COOH) of GO, thereby improving drug adsorption. Additionally, hydrogen bonding may occur between CA and the GO surface, further enhancing drug encapsulation efficiency. Beyond hydrogels, many other delivery carriers—such as nanoparticles and microspheres—can also adopt physical encapsulation. For example, one study adsorbed MTX onto glycyrrhizic acid-modified carbon dots via π–π stacking interactions to achieve drug loading [89]. In particular, for polysaccharide-based vesicles and micellar systems, the hydrophilic–lipophilic balance of drugs is often exploited to encapsulate them within hydrophobic cores or cavities. Drugs loaded via physical entrapment are typically released through diffusion or carrier degradation. However, this method may result in an initial burst release, which is undesirable in treatments requiring precise release kinetics [109].

In contrast, chemical grafting leverages the abundant active groups on polysaccharide molecules to covalently conjugate drugs to the carrier, forming stable drug–carrier complexes that allow more precise control over drug release [110]. Common strategies include the use of stimuli-responsive linkers. Researchers utilized thioxanthene (TK) as a ROS-responsive moiety to synthesize an amphiphilic PLGA-TK-PEG copolymer, which self-assembled into micelles for the co-encapsulation of the natural compounds berberine (Ber) and sinapine (Sin) [111]. These micelles were subsequently incorporated into a carboxymethyl cellulose-based soluble microneedle patch (B/S-TM@MN) to enable transdermal drug delivery. Within the high-ROS microenvironment of RA joints, the micelles underwent precise drug release through cleavage of the TK bond, concurrently exerting anti-inflammatory effects (such as promoting M2 macrophage polarization and reducing proinflammatory cytokine levels) and anti-angiogenic activity (including inhibition of endothelial cell migration and tube formation). The system demonstrated synergistic therapeutic efficacy in a CIA mouse model.

Furthermore, a combination of physical encapsulation and chemical grafting can significantly improve drug encapsulation efficiency. For example, in one study, the amphiphilic conjugate polymer DS-cad-DXM was synthesized via a two-step chemical reaction [112]. The copolymer was then co-dissolved with free dexamethasone (DXM) in dimethyl sulfoxide (DMSO), and micelles were formed through dialysis-induced self-assembly. In this system, the hydrophobic core physically entraps free DXM via hydrophobic interactions, while the hydrophilic dextran sulfate (DS) shell provides active targeting capability. The resulting DXM@DS-cad-DXM micelles had a reported drug loading of 24.3% ± 3.7%. This example illustrates that a rational combination of physical and chemical loading strategies holds significant promise for enhancing drug loading capacity.

Overall, physical entrapment is preferred for its operational simplicity and broad applicability and is suitable for most small-molecule drugs; however, it generally suffers from unavoidable initial burst release and limited loading stability. In contrast, chemical grafting offers superior release precision and sustained release performance but introduces additional synthetic steps and carries a risk of altering drug biological activity. The choice of loading method should be determined by the drug’s physicochemical properties, the required release profile, and manufacturing feasibility.

### 6.4. Carrier Type Selection

Employing distinct types of drug carriers tailored to different therapeutic cargos, administration routes, and desired release profiles represents a practical strategy for improving the performance of RA drug delivery systems. Polysaccharide-derived carriers offer diverse physicochemical and functional characteristics that can be adapted to specific therapeutic requirements, including prolonged local retention, controlled release, cellular uptake, and minimally invasive administration. Therefore, carrier selection should be based on the properties of the therapeutic cargo and the intended delivery objective rather than on a fixed assignment to a particular disease stage. Table 2 systematically summarizes key advantages, responsiveness, suitable drug types, and applicable RA treatment scenarios for four major classes of polysaccharide-based carriers: hydrogels, microspheres, microneedles, and nanoparticles.

Polysaccharide-based hydrogels are particularly attractive when prolonged local retention and sustained drug release are required. Their three-dimensional network structure can provide an injectable depot within the joint and can be engineered to respond to selected pathological stimuli while accommodating small molecules, proteins, or nucleic acids. However, prolonged retention does not necessarily translate into superior therapeutic efficacy, and hydrogel performance depends on cross-linking chemistry, degradation behavior, cargo properties, and the local inflammatory environment. Therefore, hydrogel selection should be based on the desired balance among local retention, release kinetics, therapeutic activity, and formulation stability rather than on a predefined stage of RA progression [40,113].

Polysaccharide-derived microspheres may be advantageous when high drug-loading capacity, particle-size control, and sustained or site-specific release are required. Their formulation flexibility allows the incorporation of hydrophobic small molecules and, depending on the carrier design, peptide-based cargos for local, oral, or systemic administration. However, their therapeutic performance is strongly influenced by particle size, surface properties, loading strategy, degradation behavior, and administration route. Thus, microspheres should be considered according to the physicochemical characteristics of the therapeutic cargo and the intended delivery objective rather than being assigned to a predefined stage of RA progression [79,114].

For treatment scenarios in which minimally invasive administration or improved dosing convenience is desirable, polysaccharide-based microneedles provide a transdermal delivery platform with potential for patient self-administration. Their utility for peptide- and protein-based therapeutics depends on cargo stability, dose requirements, microneedle geometry, drug-loading strategy, and skin permeability, and should therefore not be considered universal. In the context of RA, their therapeutic value should be evaluated according to pharmacokinetics, biodistribution, synovial exposure, and therapeutic efficacy rather than presumed to depend on a particular stage of disease progression [67,111].

Furthermore, polysaccharide nanoparticles show considerable promise for immunomodulation because their size, surface properties, and functional groups can be readily tuned to influence tissue distribution, cellular uptake, and drug release. However, nanoparticle accumulation in inflamed joints should not be interpreted simply as the classical enhanced permeability and retention (EPR) effect described in tumor tissues. In RA, inflammatory vascular permeability may facilitate extravasation, while local retention and cellular uptake are also influenced by inflammatory cells, extracellular matrix interactions, receptor-mediated recognition, and lymphatic clearance. Therefore, these processes are better described as inflammation-associated vascular permeability, cellular uptake, and local retention rather than as a direct equivalent of the tumor EPR effect. They are frequently designed as dual-responsive smart systems sensitive to pH and ROS, allowing targeted delivery of biological agents, including siRNA and immunomodulators, to correct aberrant immune responses and remodel the joint microenvironment [115].

In summary, carrier selection should be guided by the properties of the therapeutic cargo, administration route, desired release profile, targeting mechanism, safety considerations, and the specific pathological and therapeutic context. A rational selection strategy should focus on matching carrier characteristics with therapeutic requirements while balancing local exposure, treatment convenience, formulation complexity, and translational feasibility. This approach avoids assuming that a particular carrier type is inherently superior for a predefined stage of RA and instead emphasizes context-dependent optimization of delivery performance.

Importantly, direct head-to-head comparisons of different polysaccharide carrier architectures within the same RA model remain limited. Most available studies evaluate a single carrier design against free drug, vehicle, or untreated controls, rather than comparing two or more polysaccharide-based carriers under otherwise comparable experimental conditions. Consequently, differences in therapeutic outcomes cannot always be attributed to the carrier architecture itself, because drug loading, administration route, dose, treatment duration, disease severity, and evaluation methods may also differ among studies. Therefore, the current evidence does not support a universal hierarchy among hydrogels, nanoparticles, microspheres, microneedles, or polysaccharide-containing composite systems. Standardized head-to-head studies using the same therapeutic cargo, dose, administration route, RA model, treatment duration, and outcome measures would provide stronger evidence for rational carrier selection and facilitate meaningful cross-platform comparison.

## 7. Challenges and Future Directions

Despite their considerable potential in drug delivery applications, polysaccharide-derived carriers continue to face significant challenges and persisting limitations during the transition from research and development to commercial production. This section summarizes key issues in the development of polysaccharide-based drug delivery systems and outlines future directions, with the aim of offering valuable insights for ongoing research in this field.

### 7.1. Challenges

When designing polysaccharide-derived drug delivery carriers, several notable challenges must be considered:

Safety assessment remains a major challenge for polysaccharide-based delivery systems. Although many polysaccharides generally exhibit favorable biocompatibility, the safety profile of the final delivery system is determined not only by the parent polysaccharide but also by chemical modification, degree of substitution, cross-linking density, molecular weight, residual reagents, and the incorporation of non-polysaccharide components. In particular, injectable and nanoscale systems require evaluation of local inflammatory responses, immunogenicity, hemocompatibility, systemic toxicity, organ distribution, and degradation products in addition to routine cytocompatibility testing [116,117]. These considerations are especially relevant to multifunctional composite systems containing synthetic polymers or nanomaterials, for which degradation and clearance may differ from those of the polysaccharide component. Therefore, short-term in vitro biocompatibility alone cannot establish the long-term safety of systems intended for repeated administration.

Polysaccharides are naturally derived and exhibit structural variability. Raw materials from different batches show variations in parameters such as molecular weight and viscosity, leading to inconsistencies in the performance of the resulting drug carriers under in vivo conditions [118,119].

Synthetic complexity presents another major hurdle: optimizing polysaccharide modifications frequently involves multi-step reactions, which complicates the control over synthesis reproducibility and stability. Precise modulation of modification and crosslinking degrees is difficult, influencing critical properties like particle size uniformity and drug loading stability. These factors collectively hinder scalable production [30]. For instance, during polysaccharide sulfation, controlling the degree of substitution with precision remains particularly challenging [120].

### 7.2. Clinical Translation and Manufacturing Considerations

Most polysaccharide-derived drug delivery systems investigated for RA remain at the preclinical stage. Importantly, clinical experience with polysaccharides themselves should be distinguished from clinical validation of engineered polysaccharide-derived drug delivery systems. Hyaluronic acid (HA), for example, has been clinically investigated as an intra-articular intervention in patients with rheumatoid arthritis (RA). In a multicenter clinical trial, intra-articular sodium hyaluronate was administered weekly for five consecutive weeks to 25 patients with RA, resulting in significant improvements in pain, local inflammation, and visual analog scale scores [121]. Another clinical study involving 118 patients with RA evaluated intra-articular high-molecular-weight hyaluronan in rheumatoid knees with joint effusion, with patients followed for six months [122]. In addition, a meta-analysis of five randomized controlled trials involving 720 participants reported beneficial effects of intra-articular hyaluronate on pain and inflammation in rheumatoid knees [123]. These studies demonstrate that HA-based local intervention has been clinically investigated in RA; however, they primarily evaluated HA as a therapeutic or joint-localizing material rather than as a multifunctional drug delivery platform incorporating engineered targeting, controlled release, or stimulus-responsive mechanisms. Previous analyses of polysaccharide-based delivery systems have identified material heterogeneity, incomplete characterization, manufacturing reproducibility, and regulatory requirements as important barriers to clinical translation [124].

From a clinical perspective, local polysaccharide-based delivery should also be considered in the context of RA as a systemic autoimmune disease. Intra-articular or other locally administered systems may increase drug exposure at selected inflamed sites and potentially reduce systemic exposure; however, they cannot by themselves address polyarticular disease or extra-articular manifestations. Therefore, local polysaccharide-based delivery should be regarded as a complementary or site-specific strategy rather than a replacement for systemic disease-modifying antirheumatic therapy. Intra-articular administration may be particularly relevant for monoarticular or oligoarticular flares or for a limited number of persistently active joints despite otherwise controlled systemic disease, where local treatment can provide site-specific anti-inflammatory effects. In contrast, patients with persistent polyarticular disease, widespread joint involvement, or clinically significant extra-articular manifestations generally require systemic disease-modifying treatment, because local injection of individual joints cannot adequately control the systemic inflammatory process. In patients with a persistent polyarticular flare, local injections may be used as adjunctive treatment for symptomatic or particularly active joints but should not substitute for reassessment and optimization of systemic DMARD therapy. Future clinical studies should therefore evaluate localized delivery according to the number and distribution of actively inflamed joints, systemic disease activity, extra-articular involvement, and the feasibility of repeated local administration, and should determine whether localized delivery provides clinically meaningful advantages over established systemic treatment, including improved local efficacy, reduced systemic exposure, improved tolerability, or reduced dosing frequency.

Translation also requires control of critical formulation attributes, including molecular weight, degree of substitution, particle size, drug loading, cross-linking, sterility, and release characteristics. For nanoscale systems, reproducible control of physicochemical properties and batch-to-batch consistency becomes particularly important during scale-up, while purification, endotoxin control, storage stability, and process robustness must also be considered [125,126].

The addition of synthetic polymers, nanomaterials, targeting ligands, or multiple therapeutic cargos may provide additional functions but also increases formulation complexity and the requirements for characterization and safety assessment. Therefore, the benefit of additional functionality should be weighed against manufacturing difficulty, quality-control burden, safety risks, and production costs. Future studies should place greater emphasis on quantitative pharmacokinetics and biodistribution, repeated-dose safety, scalable manufacturing, and direct comparison with established RA therapies. These considerations will help determine whether improvements observed in preclinical models can provide a clinically meaningful advantage.

### 7.3. Future Directions

Looking ahead, the development prospects for polysaccharide-derived drug carriers remain highly promising, particularly in the treatment of RA. Polysaccharide-based drug delivery systems are expected to evolve toward greater intelligence, biomimicry, and technological integration. Key future trends can be summarized as follows:

1. Biomimetic design represents a major direction for future development. By emulating the structure and function of natural substances within joint tissues, this approach significantly enhances carrier performance [127]. Certain polysaccharides—such as HA and chondroitin sulfate—can mimic the physiological microenvironment of the native extracellular matrix. Sulfated polysaccharides like sulfated cellulose exhibit negative charge densities similar to those of sulfated glycosaminoglycan [128]. Designing carriers that imitate joint matrix components may improve drug delivery efficiency for RA therapy.
2. Stimuli-responsive systems offer a powerful strategy for achieving precision in drug delivery. Drug release is no longer limited to single stimuli; instead, integrating multiple responsive elements into carriers enables conditional release under varying microenvironments, supporting accurate spatiotemporal control throughout the treatment process [129].
3. The integration of advanced manufacturing technologies will facilitate more efficient and controllable production of polysaccharide-derived carriers. For instance, 3D printing has already been incorporated in the fabrication of polysaccharide-based microneedles [70]. Future progress may employ 3D and 4D printing to produce hydrogels, microneedles, and other carrier types, introducing capabilities such as dynamic shape transformation while enabling precise control over macro-scale geometry and micro-scale porosity [130]. The incorporation of AI and large-language models could accelerate the screening of carrier formulations and prediction of drug release profiles, thereby streamlining material design and experimental optimization [131]. Additionally, techniques such as supramolecular self-assembly provide novel avenues for constructing complex polysaccharide-based carrier architectures.

## 8. Conclusions and Outlook

As a chronic inflammatory disease with no complete cure available, a central challenge in treating RA is achieving sustained and targeted delivery of anti-inflammatory drugs to affected joints while minimizing systemic exposure and associated toxic side effects. Polysaccharides, with their diverse modification strategies, excellent biocompatibility, and biodegradability, offer significant potential and distinct advantages for drug delivery applications. This review summarizes recent advances in polysaccharide-derived drug delivery systems for RA treatment, focusing on two main aspects: the variety of carrier forms and rational design strategies. Polysaccharide-based hydrogels, nanoparticles, microneedles, and composite carriers have shown promising therapeutic effects in preclinical models of RA, with potential to improve local drug retention, controlled release, or delivery efficiency depending on the carrier architecture and therapeutic cargo. However, these findings should not be interpreted as evidence of clinical superiority over established RA therapies. Important limitations remain, including limited quantitative pharmacokinetic and biodistribution data, insufficient long-term and repeated-dose safety evaluation, incomplete manufacturing validation, and the lack of standardized comparisons among different carrier types.

Future studies should therefore prioritize clinically relevant evaluation rather than further increasing structural complexity alone. Direct comparison with existing RA treatments, quantitative assessment of tissue drug exposure, long-term safety studies, scalable and reproducible manufacturing, and well-designed translational studies will be essential to determine whether the advantages observed in preclinical models can be translated into meaningful clinical benefits. At present, polysaccharide-derived delivery systems should be regarded as promising preclinical approaches with considerable translational potential, rather than clinically validated alternatives to established RA therapies.

## Figures and Tables

**Table 2:** Selection methods for four common polysaccharide derivative drug delivery carriers in the treatment of RA.

Carrier Type	Polysaccharide Role	Core Advantages	Typical Release/Response	Administration Route	Representative RA Application	Main Limitation
Polysaccharide-based hydrogel	Matrix/depot	Injectable; prolonged retention	Sustained; pH/ROS/enzyme-responsive when engineered	Intra-articular	Local delivery of MTX, proteins, nucleic acids	Limited mechanical stability; formulation complexity
Polysaccharide-derived nanoparticles	Carrier/targeting component	High surface area; tunable size and surface	Controlled or stimulus-responsive	Systemic/local	Small molecules, gene-related cargos	Rapid clearance; targeting requires in vivo validation
Polysaccharide-based microneedles	Matrix/transdermal depot	Minimally invasive; self-administration potential	Dissolution or controlled release	Transdermal	MTX, EVs, anti-inflammatory agents	Joint exposure requires pharmacokinetic validation
Polysaccharide-derived microspheres	Drug reservoir	Sustained release; high loading potential	Diffusion/degradation-controlled	Oral/local/systemic depending on design	MTX or peptide delivery	Manufacturing and size control
Polysaccharide-containing composites	Matrix + functional module	Multifunctional; combined delivery functions	Carrier-dependent	Route-dependent	Combination therapy	Increased complexity and safety/quality-control burden

## Data Availability

No new data were created or analyzed in this study. Data sharing is not applicable to this article.

## Abbreviations

The following abbreviations are used in this manuscript:

2-DG	2-deoxy-D-glucose
ACPA	anti-citrullinated protein antibodies
AD	adamantane
AGP	aloe vera gel powder
ALG	alginate
ALG-TYR	alginate-tyramine
CA	cichoric acid
CDs	carbon dots
CHS	chondroitin sulfate
CIA	collagen-induced arthritis
COX	cyclooxygenase
CS	chitosan
CSC	chondroitin sulfate C
CSMA	methacrylated chondroitin sulfate
DDS	drug delivery systems
DIDS	disulfide cross-linker
DMSO	dimethyl sulfoxide
DS	dextran sulfate
DXM	dexamethasone
EDC	1-ethyl-3-(3-dimethylaminopropyl)carbodiimide
EVs	extracellular vesicles
FLS	fibroblast-like synoviocytes
FmocFF	fluorenylmethoxycarbonyl-phenylalanine-phenylalanine
Fuc	fucoidan
GA	glycyrrhetinic acid
GO	graphene oxide
HA	hyaluronic acid
hASC-EVs	human adipose stem cell-derived extracellular vesicles
HTDD	hyaluronic acid-triglycerol monostearate dual-drug vesicles
IA	intra-articular
IL-1β	interleukin-1 beta
IL-6	interleukin-6
IL-17	interleukin-17
JAK	Janus kinase
LDH	layered double hydroxide
LEF	leflunomide
MEL	melittin
MMPs	matrix metalloproteinases
MMP-2/9	matrix metalloproteinases-2 and -9
MMP-13	matrix metalloproteinase-13
MNs	microneedles
MLX	meloxicam
MSPs	microsponges
MTX	methotrexate
NPs	nanoparticles
NSAIDs	nonsteroidal anti-inflammatory drugs
OA	osteoarthritis
OCS	oxidized chondroitin sulfate
OHA	oxidized hyaluronic acid
PEI	polyethyleneimine
PEG	polyethylene glycol
PLGA	poly(lactic-co-glycolic acid)
PVs	polymeric vesicles
QCS	quaternized chitosan
RA	rheumatoid arthritis
RF	rheumatoid factor
ROS	reactive oxygen species
SAN	single-atom nanozyme
SEM	scanning electron microscopy
SIN	sinomenine
SR-A	scavenger receptor class A
TGMS	triglycerol monostearate
TK	thioketone
TNF-α	tumor necrosis factor-alpha
TPL	triptolide
UV–Vis	ultraviolet–visible spectroscopy
